# Prognostic Factors for Excellent Response to Initial Therapy in Patients With Papillary Thyroid Cancer From a Prospective Multicenter Study

**DOI:** 10.3389/fonc.2022.840714

**Published:** 2022-07-04

**Authors:** Wen-Wu Dong, Da-Lin Zhang, Liang He, Liang Shao, Zhi-Hong Wang, Cheng-Zhou Lv, Ping Zhang, Tao Huang, Hao Zhang

**Affiliations:** ^1^ Department of Thyroid Surgery, The First Hospital of China Medical University, Shenyang, China; ^2^ Department of Breast and Thyroid Surgery, Wuhan Union Hospital, Tongji Medical College, Huazhong University of Science and Technology, Wuhan, China

**Keywords:** response to therapy, prognostic factors, papillary thyroid cancer, thyroglobulin, extrathyroidal extension

## Abstract

Prognostic factors for excellent response (ER) to initial therapy in patients with papillary thyroid cancer (PTC) have not been determined. In this study, we investigated the response to initial therapy in PTC patients and independent prognostic factors for ER in a prospective multicenter study in China. A total of 506 PTC patients from nine centers in China were enrolled in this study, all of whom underwent total or near total thyroidectomy with lymph node dissection and subsequent radioiodine therapy. Univariate and multivariable logistic regression analyses were carried out to determine the independent prognostic factors for ER. The optimal cutoff value of the number of metastatic lymph nodes for predicting ER was determined by the receiver operating characteristic curve. A total of 139 patients (27.5%) achieved ER after initial therapy. Extrathyroidal extension, the number of metastatic lymph nodes, and preablative-stimulated thyroglobulin (Ps-Tg) were independent risk factors for ER for the entire population. In a subgroup analysis, extrathyroidal extension and Ps-Tg were independent risk factors for ER in pathological N1a patients, while the number of metastatic lymph nodes and Ps-Tg were independent risk factors for ER in pathological N1b patients. The appropriate cutoff values of the number of metastatic lymph nodes in predicting ER were 5 and 13 for the entire population and pathological N1b PTC patients, respectively. PTC patients with more metastatic lymph nodes were more likely to fail to achieve ER. Extrathyroidal extension, the number of metastatic lymph nodes, and Ps-Tg were important prognostic factors for ER after initial therapy in PTC patients.

## Introduction

Papillary thyroid cancer (PTC) is the most common endocrine neoplasm and the most frequent malignant thyroid tumor. While thyroid cancer incidence increased for several years, it has now plateaued or even started to decrease (1, 2). Most patients with PTC have an excellent prognosis with an extremely low disease-specific mortality rate after optimized treatment but may suffer a recurrence even 10 or more years after initial treatment. Further, the risk of disease-specific mortality and recurrence is not immutable but can change over time with the clinical course of the disease and the response to initial therapy for an individual patient (3, 4). Thus, the 2015 revised American Thyroid Association (ATA) guidelines has proposed dynamic risk stratification (DRS), using a new therapeutic response system to guide the individualized management of differentiated thyroid cancer (DTC) patients and specify the mortality and recurrence in patients after both total/near total thyroidectomy and radioiodine (RAI) therapy, which appears to be a more objective ongoing assessment in predicting the clinical outcome. The DRS system classified patients into four response-to-therapy categories on the basis of clinical, biochemical, and imaging (structural and functional), and cytopathologic findings obtained during the first 2 years of follow-up, including excellent, biochemical incomplete, structural incomplete, and indeterminate. Therefore, it is critical to identify the predictors for the response to therapy. Previous studies showed some clinicopathological features (e.g., sex, tumor size, lymphovascular invasion, number of metastatic lymph nodes [LNs], ratio of metastatic to examined LNs [LNR], extranodal extension, and preablative-stimulated thyroglobulin [Ps-Tg]) may affect the response to therapy (5–11). However, these results are based on retrospective single-center studies, and some of them are inconsistent. For example, some studies suggested that lymph node metastasis was an independent risk factor for non-excellent response to initial therapy, but other studies found no relationship between lymph node metastasis and response to initial therapy (5, 8–10). Thus, the primary aim of this study was to identify prognostic factors for excellent response (ER) in PTC patients in a prospective multicenter study.

## Materials and Methods

### Study Design

A prospective multicenter study to observe the initial management of patients with DTC in the real world from nine hospitals in China (DTCC study) was launched. The trial is registered at ClinicalTrials.gov (NCT02638077). Patients who were diagnosed with DTC after initial surgical treatment were included in the DTCC study and followed up for at least 1 year. A total of 2,013 patients with DTC who underwent thyroidectomy with lymph node dissection between October 2014 and July 2016 were enrolled. Among them, 506 patients were included in this study. The inclusion criterion included PTC patients who underwent total or near total thyroidectomy with lymph node dissection and subsequent RAI therapy and pathologically confirmed lymph node metastasis. Exclusion criteria were a history of previous thyroidectomy, presence of distant metastasis at the initial presentation, insufficient medical records, or inadequate follow-up information. This study was approved by the Institutional Review Boards of each center prior to patient enrollment and conducted in accordance with the Declaration of Helsinki. Written informed consent was obtained from all participants.

### Initial Therapy

Initial therapy was defined as the initial surgical procedure, TSH suppression therapy, and the first RAI therapy. Total or near total thyroidectomy was the primary surgical procedure and usually performed when any of the following conditions was met: (a) primary tumor >4 cm; (b) bilateral or multiple lesions; (c) extrathyroid extension; (d) clinical evidence of lymph node metastasis or distant metastasis. A central lymph node dissection (CND), at least ipsilateral CND, was routinely performed for all the patients. Bilateral CND was performed for bilateral carcinoma or due to a clinically suspicious nodal disease in the area of the contralateral central compartment. Lateral neck dissection (LND), including levels II–V, was performed only in cases with clinically suspicious or pathologically proven metastatic lateral neck lymph nodes. All patients took levothyroxine (LT4) for TSH suppression therapy after surgery, and a dose of LT4 was delivered according to recurrence risk stratification. All patients received RAI therapy following LT4 withdrawal, accompanied by a low-iodine diet for at least 2 weeks. A dose of RAI ranging from 1.11 GBq (30 mCi) to 7.40 GBq (200 mCi) was administered according to the American Joint Committee on Cancer (AJCC) TNM staging system and recurrence risk stratification. Routine biochemical examinations (e.g., serum thyrotropin [TSH], Ps-Tg, anti-Tg antibody [TgAb]) were measured, and imaging examinations (e.g., ultrasonography, computed tomography, or diagnostic RAI whole-body scan, if necessary) were performed before RAI therapy. After RAI therapy, LT4 was administered to all patients for TSH suppression therapy.

### Follow-Up Protocol

The follow-up strategy was mainly based on the latest ATA guidelines. Instructed and trained professional staff members were responsible for the follow-up process at each center, and data of the follow-up were recorded in case report form. Generally, the follow-up periods were 1 month, 3 months, 6 months, and 1 year after operation and annually thereafter. All patients were regularly followed by physical, biochemical, and imaging examinations. Long-term monitoring of patients with DTC was guided by the patients’ response to therapy during the first year of follow-up. Thus, response to initial therapy at 1 year after operation was evaluated according to the reclassification system proposed by the latest ATA guidelines.

### Statistical Analysis

Continuous and categorical variables are expressed as median (interquartile range) and numbers (percentages), respectively. Univariate and multivariable logistic regression analyses were employed to determine the independent prognostic factors for ER, including age at surgery, sex, tumor size, extrathyroidal extension, multifocality, N status, number of LNs retrieved, number of metastatic LNs, LNR of LNs, Ps-Tg, time interval between surgery and RAI, and dose of RAI therapy. Odds ratios [ORs] were presented with their 95% confidence intervals (CIs). The optimal cutoff value of the number of metastatic LNs for predicting ER was determined by the receiver operating characteristic (ROC) curve. All statistical analyses were performed using the Stata Statistical Software Package 9.0 (Stata Corporation Ltd., College Station, TX, USA). All statistical tests were two sided, and differences were considered statistically significant if a *P* value < 0.05.

## Results

### Patient Characteristics

The detailed clinical characteristics of the study population are summarized in **Table 1**. Unilateral CND was performed in 113 (22.3%) patients and bilateral CND in 393 (77.7%) patients. Unilateral LND was performed in 202 (39.9%) patients and bilateral LND in 32 (6.3%) patients. A total of 294 (58.1%) patients had pathological N1a (pN1a) disease, whereas the remaining 212 (41.9%) patients had pathological N1b (pN1b) disease. The mean numbers of positive and total lymph nodes were 4.43 ± 3.99 (range 0–29) and 11.60 ± 7.17 (range 0–46) in CND, and 6.58 ± 6.51 (range 0–52) and 23.27 ± 20.19 (range 1–160) in CND+LND, respectively. After initial treatment, 139 (27.5%) patients with PTC presented ER. No patient died of PTC. Recurrence occurred in four (0.8%) patients, all of which were located in regional LNs. Distant metastasis to the lung occurred in one (0.2%) patient.

**Table 1:** Clinicopathologic characteristics of patients with papillary thyroid cancer by response to initial therapy.

Patient characteristics	ER, n=139 (%)	Non-ER, n=367(%)
Age at surgery (years)		
< 55	129 (92.8)	326 (88.8)
≥ 55	10 (7.2)	41 (11.2)
Sex		
Male	47 (33.8)	122 (33.2)
Female	92 (66.2)	245 (66.8)
Tumor size (cm)		
≤4	134 (96.4)	343 (93.5)
>4	5 (3.6)	24 (6.5)
Exe		
No	90 (64.7)	195 (53.1)
Yes	49 (35.3)	172 (46.9)
Multifocality		
No	79 (56.8)	213 (58.0)
Yes	60 (43.2)	154 (42.0)
N status		
N1a	88 (63.3)	206 (56.1)
N1b	51 (36.7)	165 (43.9)
No. of LNs retrieved	16 (8-28)	19 (9-34)
No. of metastatic LNs	4 (2-7)	5 (2-10)
LNR of LNs	0.27 (0.14-0.50)	0.29 (0.17-0.47)
Ps-Tg (ng/mL)		
≤2	111 (79.9)	190 (51.8)
>2	28 (20.1)	177 (48.2)
Time interval between surgery and RAI (month)		
<3	52 (37.4)	158 (43.1)
≥3	87 (62.6)	209 (56.9)
Initial dose of RAI (mCi)		
<100	10 (7.2)	26 (7.1)
≥100	129 (92.8)	341 (92.9)

ER, excellent response; Exe, Extrathyroidal extension; LNs, lymph nodes; LNR, ratio of metastatic to examined lymph nodes; Ps-Tg, preablative-stimulated thyroglobulin; RAI, radioiodine.

### Prognostic Factors for ER for the Entire Population

Univariate analysis indicated that extrathyroidal extension (*P* = 0.019), number of metastatic LNs (*P* = 0.002), and Ps-Tg level (*P* < 0.001) were significantly related to ER in this cohort (**Table 2**). Age at surgery, sex, tumor size, multifocality, N status, number of LNs retrieved, LNR of LNs, initial dose of RAI therapy, and time interval between surgery and RAI therapy did not demonstrate significant between-group differences (**Table 2**). Furthermore, multivariable analysis also showed that extrathyroidal extension (OR = 1.644; 95% CI: 1.082–2.500; *P* = 0.020), number of metastatic LNs (OR = 1.042; 95% CI: 1.001–1.083; *P* = 0.042), and Ps-Tg level (OR = 3.317; 95% CI: 2.065–5.329; *P* < 0.001) were independent prognostic factors for ER (**Table 2**).

**Table 2:** Univariate and multivariable analyses of prognostic factors for excellent response for the entire population.

Variable	Univariate analysis		Multivariate analysis	
	OR (95% CI)	P	OR (95% CI)	P
Age at surgery (<55 years vs. ≥55 years)	1.622 (0.789-3.335)	0.188		
Sex (Female vs. Male)	0.975 (0.645-1.473)	0.903		
Tumor size (≤4 cm vs. >4 cm)	1.875 (0.701-5.016)	0.210		
Exe (negative vs. positive)	1.620 (1.082-2.426)	0.019	1.644 (1.082-2.500)	0.020
Multifocality (negative vs. positive)	0.952 (0.642-1.412)	0.807		
N status (N1a vs. N1b)	1.349 (0.902-2.016)	0.145		
No. of LNs retrieved	1.008 (0.997-1.019)	0.149		
No. of metastatic LNs	1.064 (1.023-1.107)	0.002	1.042(1.001-1.083)	0.042
LNR of LNs	1.543 (0.650-3.666)	0.326		
Ps-Tg (≤2 ng/mL vs. >2 ng/mL)	3.693 (2.326-5.864)	<0.001	3.317(2.065-5.329)	<0.001
Initial dose of RAI (<100 mCi vs.≥100 mCi)	1.017 (0.477-2.167)	0.966		
Time interval between surgery and RAI (<3 months vs. ≥3 months)	0.791 (0.529-1.181)	0.251		

Exe, Extrathyroidal extension; LNs, lymph nodes; LNR, ratio of metastatic to examined lymph nodes

Ps-Tg, preablative-stimulated thyroglobulin; RAI, radioiodine; OR, odds ratio; CI, confidence interval.

### Prognostic Factors for ER for pN1a PTC Patients

For this part of patients, univariate analysis indicated that extrathyroidal extension (*P* = 0.013) and Ps-Tg level (*P* < 0.001) significantly correlated with ER (**Table 3**). Age at surgery, sex, tumor size, multifocality, number of LNs retrieved, number of metastatic LNs, LNR of LNs, initial dose of RAI therapy, and time interval between surgery and RAI therapy did not correlate with ER (**Table 3**). Furthermore, multivariable analysis also showed that extrathyroidal extension (OR = 2.039; 95% CI: 1.178–3.528; *P* = 0.011) and Ps-Tg level (OR = 3.439; 95% CI: 1.836–6.441; *P* < 0.001) were independent prognostic factors for ER (**Table 3**).

**Table 3:** Univariate and multivariable analyses of prognostic factors for excellent response for pN1a PTC patients.

Variable	Univariate analysis		Multivariate analysis	
	HR (95% CI)	P	HR (95% CI)	P
Age at surgery (<55 years vs. ≥55 years)	1.314 (0.537-3.213)	0.550		
Sex (Female vs. Male)	1.056 (0.619-1.802)	0.842		
Tumor size (≤4 cm vs. >4 cm)	5.860 (0.755-45.504)	0.091		
Exe (negative vs. positive)	1.963 (1.151-3.347)	0.013	2.039 (1.178-3.528)	0.011
Multifocality (negative vs. positive)	0.835 (0.498-1.400)	0.495		
No. of LNs retrieved	1.000 (0.972-1.029)	0.992		
No. of metastatic LNs	1.074 (0.973-1.187)	0.157		
LNR of LNs	1.362 (0.510-3.635)	0.538		
Ps-Tg (≤2 ng/mL vs. >2 ng/mL)	3.351 (1.800-6.237)	<0.001	3.439 (1.836-6.441)	<0.001
Initial dose of RAI (<100 mCi vs.≥100 mCi)	0.593 (0.214-1.642)	0.314		
Time interval between surgery and RAI (<3 months vs. ≥3 months)	0.668 (0.400-1.116)	0.123		

Exe, Extrathyroidal extension; LNs, lymph nodes; LNR, ratio of metastatic to examined lymph nodes;

Ps-Tg, preablative-stimulated thyroglobulin; RAI, radioiodine; OR, odds ratio; CI, confidence interval.

### Prognostic Factors for ER for pN1b PTC Patients

For these patients, number of metastatic LNs (*P* = 0.011) and Ps-Tg (*P* < 0.001) were associated with ER (**Table 4**). Age at surgery, sex, tumor size, extrathyroidal extension, multifocality, number of LNs retrieved, LNR of LNs, initial dose of RAI therapy, and time interval between surgery and RAI therapy did not show significant associations with ER (**Table 4**). Furthermore, multivariable analysis also showed that number of metastatic LNs (OR = 1.050; 95% CI: 0.995–1.107; *P* = 0.044) and Ps-Tg level (OR = 3.487; 95% CI: 1.699–7.157; *P* < 0.001) were independent prognostic factors for ER (**Table 4**).

**Table 4:** Univariate and multivariable analyses of prognostic factors for excellent response for pN1b PTC patients.

Variable	Univariate analysis		Multivariate analysis	
	OR (95% CI)	P	OR (95% CI)	P
Age at surgery (<55 years vs. ≥55 years)	2.270 (0.646-7.976)	0.201		
Sex (Female vs. Male)	0.850 (0.441-1.637)	0.627		
Tumor size (≤4 cm vs. >4 cm)	0.862 (0.262-2.833)	0.806		
Exe (negative vs. positive)	1.173 (0.623-2.207)	0.621		
Multifocality (negative vs. positive)	1.023 (0.545-1.921)	0.943		
No. of LNs retrieved	1.006 (0.991-1.022)	0.427		
No. of metastatic LNs	1.071 (1.016-1.129)	0.011	1.050 (0.995-1.107)	0.044
LNR of LNs	3.453 (0.539-22.127)	0.191		
Ps-Tg (≤2 ng/mL vs. >2 ng/mL)	3.998 (1.979-8.076)	<0.001	3.487 (1.699-7.157)	<0.001
Initial dose of RAI (<100 mCi vs.≥100 mCi)	2.391 (0.725-7.892)	0.152		
Time interval between surgery and RAI (<3 months vs. ≥3 months)	1.004 (0.526-1.913)	0.991		

Exe, Extrathyroidal extension; LNs, lymph nodes; LNR, ratio of metastatic to examined lymph nodes;

Ps-Tg, preablative-stimulated thyroglobulin; RAI, radioiodine; OR, odds ratio; CI, confidence interval.

### Identification of Appropriate Cutoff Values of the Number of Metastatic LNs in Predicting ER for the Entire Population and pN1b PTC Patients

**Figure 1:**
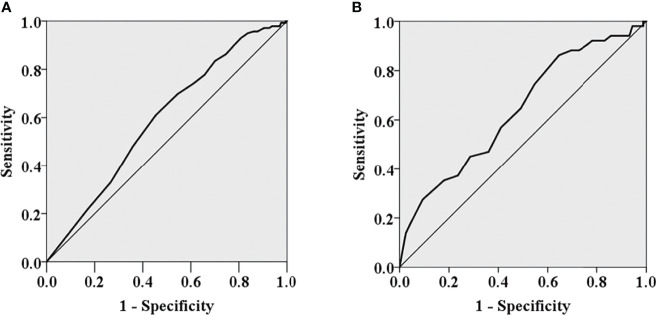
The ROC curves of the number of metastatic lymph nodes for predicting excellent response for the entire population **(A)** and pN1b PTC patients **(B)**.

The appropriate cutoff value of the number of metastatic LNs in predicting ER for the entire population was 5 (area under the ROC curve, 0.591; SE, 0.027; *P* = 0.002) with a sensitivity of 61.2% and a specificity of 54.5%, respectively (**Figure 1**). PTC patients with ≤5 metastatic LNs were more likely to obtain ER than those with >5 metastatic LNs (*P* < 0.05). 13 was determined as the appropriate cutoff value of metastatic LNs for prediction of ER in pN1b PTC patients (area under the ROC curve, 0.639; SE, 0.045; *P* = 0.003) with a sensitivity of 86.3% and a specificity 35.4%, respectively (**Figure 1**). pN1b PTC patients with ≤13 metastatic LNs were more likely to obtain ER than those with >13 metastatic LNs (*P* < 0.05).

## Discussion

In clinical practice, patients with aggressive features are more likely to fail to achieve ER and eventually experience adverse oncologic events. The clinicians want to understand whether the cause of this consequence is due to the clinicopathological characteristics of patients themselves or the inappropriate preparation process of RAI therapy. Several studies were carried out to explore the potential prognostic factors for ER, but there was no consensus. In this prospective multicenter study, we identified that extrathyroidal extension, number of metastatic LNs, and especially Ps-Tg were important prognostic factors for ER after initial therapy in PTC patients.

Extrathyroidal extension is considered as a prominent prognostic factor for PTC, especially extensive extrathyroidal extension. Extensive extrathyroidal extension has been found to be associated with recurrence in PTC patients, but minimal extrathyroidal extension has little prognostic value (12). Yang et al. found that extrathyroidal extension was associated with a structural incomplete therapeutic response in DTC patients (5). Sung et al. reported that extensive extrathyroidal extension, but not minimal extrathyroidal extension, was an independent risk factor associated with non-excellent response to initial therapy in patients with stage I classical PTC younger than 45 years (8). Lee et al. obtained the same results as Sung et al. did in patients with PTC measuring 1 to 4 cm (10). Lang et al. reported that microscopic extrathyroidal extension did not correlate with incomplete response for 1- to 4-cm PTC patients without preoperatively or intra-operatively high-risk features (e.g., radiation history, clinical nodal metastasis, and distant metastasis) (7). In our study, we found that extrathyroidal extension was an independent prognostic factor for ER for the entire population and pN1a PTC patients, even though we did not distinguish between extensive and minimal extrathyroidal extension.

The number of metastatic LNs is well-recognized as an important parameter for predicting PTC recurrence in the ATA risk stratification system. However, its predictive value for the response to initial therapy remains to be resolved. Sung et al. reported that the number of metastatic LNs could be a potentially significant risk factor for non-excellent response to initial therapy in stage I PTC patients younger than 45 years (8). Lee et al. further identified the number of metastatic LNs greater than 2.0 as an independent risk factor for non-excellent response to initial therapy in PTC measuring 1 to 4 cm (10). Gao et al. found that the number of metastatic LNs was demonstrated to be an independent predictive factor for ER in PTC patients with >10 dissected LNs (13). Our results were consistent with the above results, and the cutoff value for predicting ER to initial therapy was further determined. The appropriate cutoff values of the number of metastatic lymph nodes in predicting ER were 5 and 13 for the entire population and pathological N1b PTC patients, respectively. The prognostic value of lymph node yield in CND and LND in patients with PTC has been explored in previous studies (14–16). A higher lymph node yield in CND and LND is considered to be associated with lower disease recurrence rates. The optimal cutoff value of lymph node yield for recurrence was 11 (15). However, we found no association between lymph node yield and recurrence, possibly due to the limited follow-up time.

Thyroglobulin, a large glycoprotein secreted both by normal thyroid tissue and by thyroid cancer cells is believed to be a useful predictor of persistent or recurrent disease as well as successful ablation of the thyroid remnant and universally recommended in the follow-up of all patients with DTC. A meta-analysis indicated that Ps-Tg was a feasible tool for predicting subsequent disease-free status with high negative predictive value and 10 ng/ml was determined as the cutoff value of Ps-Tg by a summary ROC (17). Ps-Tg value >10 ng/ml increased the probability of persistent or recurrent disease, presence of distant metastases, I-131 ablation failure, and mortality (9, 18). However, whether Ps-Tg could indicate therapeutic response needs to be further outlined. Some studies reported that Ps-Tg could predict structural incomplete or indeterminate response to initial therapy (5, 11). Yang et al. found that Ps-Tg was an independent predictive factor for structural incomplete to initial therapy for the first time and 26.75 ng/ml was determined as the cutoff value of Ps-Tg by the ROC curve (5). Jeong et al. found that rhTSH-stimulated Tg measured before RAI therapy was an independent risk factor for indeterminate response to initial therapy (11). Other studies reported the value of Ps-Tg in predicting ER to initial therapy (6, 9, 13). Lawal et al. found a negative correlation between Ps-Tg and ER, although its effect did not reach a level that is statistically significant in a multivariate analysis (6). Zhang et al. found that Ps-Tg was an independent predictor for ER in the patients with Ps-Tg less than 2 ng/ml (9). Gao et al. found that Ps-Tg was an independent negative indicator for ER whether the number of dissected LNs was more than 10 or not (13). Our data also supported that Ps-Tg was a useful predictor for ER regardless of the extent of lymph node metastasis.

Although it is a prospective multicenter study, there are still some shortcomings in this study. First, the sample size of this study was relatively small and follow-up was relatively short. Second, there is little difference in surgical treatment, dose and time of RAI treatment, time of Ps-Tg detection, and postoperative management among different centers. Third, we did not consider the influence of location of metastatic LNs when we analyzed the number of metastatic LNs and LNR. Fourth, low-risk disease was excluded, and our results cannot be generalizable to all patients with PTC. Fifth, some aggressive variants (e.g., tall cell variant, columnar cell variant, and hobnail variant) of PTC and molecular mutations (e.g., BRAF V600E and TERT promoter mutations) have been associated with structural incomplete responses and higher recurrence rate (19–22). Because some institutions in this study do not routinely report PTC variants, we cannot get enough data for analysis. Thus, a well-designed prospective multicenter study with a large sample size may be needed to validate the current results.

## Conclusion

In conclusion, our study provides evidence that PTC patients with some kind of clinicopathological feature (e.g., extrathyroidal extension, more metastatic LNs, and higher Ps-Tg) are not likely to get ER. Moreover, the predictive value of these features for initial therapy outcome could be related to the extent of lymph node metastasis of PTC. With such information available, individual postoperative management and surveillance strategies will be able to be developed more easily.

## Data Availability Statement

The raw data supporting the conclusions of this article will be made available by the authors, without undue reservation.

## Ethics Statement

The studies involving human participants were reviewed and approved by the Institutional Review Boards of each center prior to patient enrollment and conducted in accordance with the Declaration of Helsinki. Written informed consent to participate in this study was provided by the participants’ legal guardian/next of kin.

## Author Contributions

Conceptualization: W-WD, TH, and HZ. Study design, data acquisition, quality control of data and algorithms: D-LZ, LH, LS, Z-HW, C-ZL, and PZ. Methodology: W-WD. Writing—original draft preparation, W-WD. Writing—review and editing, W-WD, TH, and HZ. Funding acquisition, TH. All authors contributed to the article and approved the submitted version.

## Funding

This study received funding from Merck Serono Co., Ltd., China (an affiliate of Merck KGaA Darmstadt, Germany): Grants/Research Support (No. China.DTCC.1.03).

## Conflict of Interest

The authors declare that the research was conducted in the absence of any commercial or financial relationships that could be construed as a potential conflict of interest.

## Publisher’s Note

All claims expressed in this article are solely those of the authors and do not necessarily represent those of their affiliated organizations, or those of the publisher, the editors and the reviewers. Any product that may be evaluated in this article, or claim that may be made by its manufacturer, is not guaranteed or endorsed by the publisher.
